# Current trends in global nursing: A scoping review

**DOI:** 10.1002/nop2.938

**Published:** 2021-05-22

**Authors:** Hiroko Yatsu, Akari Saeki

**Affiliations:** ^1^ Department of Nursing JIKEI University School of Medicine Tokyo Japan; ^2^ SEIBO Hospital Tokyo Japan

**Keywords:** environmental health, global health, global nursing, integrative literature review, nursing education, scoping review

## Abstract

**Aim:**

This review aimed to elucidate research trends in global nursing in international literature.

**Design:**

A scoping literature review of the PRISMA was used to guide the review.

**Methods:**

PubMed was used to search for English articles published in academic journals between 2016–2018. The search keywords were “global/international/world nursing.” We used thematic synthesis to analyse and interpret the data and generated topics for global nursing literature.

**Results:**

In total, 133 articles were analysed. Six topics emerged: (a) conceptualization of global nursing, (b) environmental health, (c) infectious diseases, (d) security efforts, (e) global shortage of nursing personnel and (f) diversification of study abroad programmes. The results of this review reflect today's serious international health, labour and global environmental issues. Based on these latest global nursing topics, it is necessary to develop new strategies, nursing models and environment‐related theories to create and maintain a healthy environment.

## 1 INTRODUCTION

Since the latter half of the 1970s, capital flow, people's movement and technology transfer based on free trade have accelerated drastically. This secondary globalization has negatively impacted the natural and social environment worldwide, such as atmospheric pollution, global warming, water and food shortages, increases in the frequency and severity of natural disasters, epidemics of infectious diseases and increasing financial disparity. Simultaneously, it has severely impacted individuals' health and well‐being globally (Edmonson et al., 2017; Goodman, 2016; Koplan et al., 2009).

The concept of global health has been established under these circumstances. The concept refers to tackling healthcare disparities to solve issues associated with regional health care, which requires collaboration beyond national and regional borders to identify health problems and plan and intervene (Edmonson et al., 2017). The concept of global nursing practice was proposed in 2000 when nursing experts began to participate in global health. Global nursing education was initiated as undergraduate and graduate programmes, mainly in Europe and North America (Wong et al., 2015).

Examples of problems that require cross‐border and cross‐regional cooperation include large‐scale storm and flood damage and fires associated with climate change and adverse effects on human health and safe living due to rising sea levels and reduced freshwater (Nicholas & Breakey, 2017). Recently, the spread of zoonotic diseases, such as coronavirus disease 2019 (COVID‐19) (Aggarwal et al., 2000; Mackenzie & Smith, 2020), has also become severe. Under these circumstances, future global nursing needs support and care from an international perspective, as well as efforts to address healthy environmental problems from a global perspective (Kleffel, 1996).

In Japan, the new curriculum implemented by the Ordinance of the Ministry of Health and the Ministry of Education amended the ordinance of 1997, which clearly stated that “in an international society, nurses [should] consider collaboration with various countries based on a wide perspective” (Hiraoka & Yoshino, 2002, p. 3). In 2006, 40% of nursing universities offered courses on global nursing practices and health care (Yoshino, 2006, p. 20). However, many Japanese research articles in global nursing practice were on “healthcare cooperation,” focusing on medical support for developing countries. There have not been many research articles based on global nursing practices (Hiraoka & Yoshino, 2002). Is the lack of research from a global perspective unique to Japan, or is it a universal problem? A review is needed to clarify research pieces conducted worldwide regarding global nursing to answer this question.

Based on the definition of Edmonson et al. (2017), this study defined global nursing as nursing in which the nursing profession participates in the extraction, planning and intervention of global health problems. Clarifying trends in the global nursing literature through this study was considered to provide a source of information for guiding nursing education, research and theory towards resolving global health problems.

## 2 BACKGROUND

The number of articles on global nursing has increased rapidly since 2000. Searching for articles in the PubMed database using the search term “global nursing” on 13 December 2020, the number of papers published since 2000 has been more than three times that of the previous 40 years. Specifically, this rapid increase in the number of articles has been remarkable since 2010. The number of papers published between 2010–2018 accounts for approximately 72% of the number of papers published since 2000.

The outline will be explained below, focusing on the literature from 2010 onwards. Research and recommendations have been made in various fields, such as geriatric nursing, midwifery care and disaster nursing. According to the World Health Organization and Alzheimer’s Disease International (2012), more than 35 million people worldwide have dementia. It is estimated that by 2050, there will be an increase of one million as the world's population ages. Tang (2014) emphasized the importance of dementia awareness training for healthcare professionals in response to this global trend. Atabay et al. (2015) reviewed workplace breastfeeding legislation in 193 member states of the United Nations.

Consequently, they found that progress in the number of countries guaranteeing breastfeeding breaks was still modest, noting the need for global efforts to promote working mothers' breastfeeding capacity. Ryan and Dogbey (2015) reported that as of 2012, 15 million preterm births were registered, many of which occurred in resource‐poor countries that lacked nursing care—India, Nigeria, Pakistan and Congo—which can be avoided. They pointed out that global action by sharing knowledge and technology needed for the millennium development goals was essential to end premature birth. Yan et al. (2015) surveyed Chinese nurses on the skills, knowledge and attitudes of disaster nursing required for earthquake relief. Consequently, all nurses believed there was a significant gap between knowledge and skills and supported the need for future disaster nursing courses. Wong et al. (2015) analysed reports published in six WHO areas between 2007–2012. The results indicated that the global level's key nursing issues were the workforce, the impact of nursing in health care, professional status and nurses' education.

These treatises have a global perspective on local issues. Many papers have pointed out that nursing problems, which initially seem to be a problem‐specific to that country or region, are a global concern that other countries or regions also have. The causes of health problems at various health levels and life stages were analysed as global problems that transcended national and regional areas. Improvement measures were considered from a global perspective.

In September 2015, the United Nations announced the sustainable development goals (SDGs). Globally, nurses and midwives provide 80% of healthcare services (WHO, 2016), and their work is critical to achieving universal health and the SDGs (Gresh et al., 2017). Although the SDGs are thought to impact nursing science significantly, few studies have revealed global nursing papers' topics based on the SDGs' focus. Therefore, in this research, we focused on articles published between 2016–2018 and attempted to clarify their characteristics.

## 3 METHOD

### 3.1 Research design

This study aimed to elucidate the trends in global nursing in international literature. The review question was as follows: What are the focus topics on global nursing papers?

A scoping review was selected to review the literature because it maps relevant published literature in a defined area—in this review, global nursing. Scoping studies have been designed to address a broader range of topics than systematic reviews. This is because they do not typically focus on a well‐defined question in which an appropriate study design can be identified in advance (Arksey & O’Malley, 2005). Scoping research aims to map the key concepts that rapidly underpin a research area and the primary sources and types of evidence available (Mays et al., 2001). Arksey and O'Malley described four motives to complete a scoping review: “To examine the extent, range, and nature of research activity”; “To determine the value of undertaking a full systematic review”; “To summarise and disseminate research findings”; and “To identify research gaps in the existing literature. (p. 21).” This study was motivated by all these possibilities, especially the first and third reasons; therefore, it was considered that there were sufficient reasons to adopt a scoping review. Critical appraisal of the literature was not undertaken in this scoping review. This is because scoping reviews do not assess the quality of evidence. Consequently, it is impossible to determine whether the reviewed papers provide robust or generalizable findings (Arksey & O’Malley, 2005, p. 27).

### 3.2 Search strategy

Preferred Reporting Items for Systematic Reviews and Meta‐Analysis (PRISMA) was used to guide the literature review. Initially, three electronic databases, PubMed (MEDLINE and PreMEDLINE), CINAHL and Ovid Nursing Database, were used to search the literature, but PubMed (MEDLINE and PreMEDLINE) was used. The reason why we used only one database is related to the purpose of this scoping review, which is to clarify the research trends of global nursing found in the international literature needed to cover a wide range of literature related to global nursing. The three databases extracted 562, 180 and 2562 documents, respectively (search date: 21 June 2019), but it was challenging to compare those documents, find duplicates and exclude them from creating a single list. Therefore, we decided to use one database, PubMed (MEDLINE and PreMEDLINE). Consequently, there was enough time to review the extracted literature; however, the provided information's completeness may be limited.

### 3.3 Inclusion and exclusion criteria

Literature was included if it met all of the following criteria: (a) extracted with the search term “global nursing,” (b) published in English between 2016–2018, and (c) the title or abstract contained one or more words of “global,” “international” or “world.” Articles were excluded if the authors did not mention global awareness and/or research objectives in the research methods and discussions section. For example, if the author set childhood obesity as a global problem, but the data were collected and analysed in only one region and the results were interpreted without comparison to global data, then the treatise was excluded from this review. No particular study design was excluded, including both qualitative and quantitative studies. Grey literature, such as government publications, non‐academic treatises and white papers, were also not excluded to understand global nursing trends better.

### 3.4 Screening

One of the two reviewers searched for literature that met criteria (a) and (b), after which the two reviewers independently screened the title and summary of the literature for (c). Next, the abstracts of the screened articles were read by two reviewers using the exclusion criteria. If one or more of the reviewers could not discern whether the document met the exclusion criteria, the two reviewers read the document's full text. There were five documents in which the two reviewers disagreed on inclusion in the analysis; thus, those documents were excluded. The two reviewers subsequently agreed to include all relevant articles (*N* = 133).

### 3.5 Data extraction

The information of the 133 target documents was converted into data using the following procedure: one reviewer extracted the data for each study using a structured form developed by the researcher; another reviewer confirmed the extracted data. The extracted data included the author name of the selected document, author affiliation information (country of affiliation, occupation, position), year of publication, volume page of the journal, article title, background and research purpose, study design, sample size, participant characteristics, data collection/analysis methods, results and discussion.

### 3.6 Data analysis

We conducted thematic synthesis based on Thomas and Harden (2008) to answer this scoping review's research question. There are three stages in the synthesis: the free line‐by‐line coding of the findings of primary literature; the organization of these “free codes” into related areas to construct “descriptive” themes; and the development of “analytical” themes. The first and second stages remain “close” to the primary study. Conversely, the third stage is an interpretation stage in which the reviewers “beyond” the primary research to generate new interpretive constructs, explanations or hypotheses (pp. 4–7).

In developing the theme of this research, each reviewer first performed line‐by‐line coding and descriptive theme generation. Next, two reviewers discussed, inferred and integrated the patterns that appear in those themes to generate six categories as themes as interpretations beyond the original literature's content. Furthermore, we derived concepts that more clearly show each theme and identified them as six topics representing recent trends in the global nursing literature.

This study was conducted without ethical approval because a literature review did not require ethical approval according to the institution's research guidelines to which the first author belongs.

## 4 RESULTS

**FIGURE 1:**
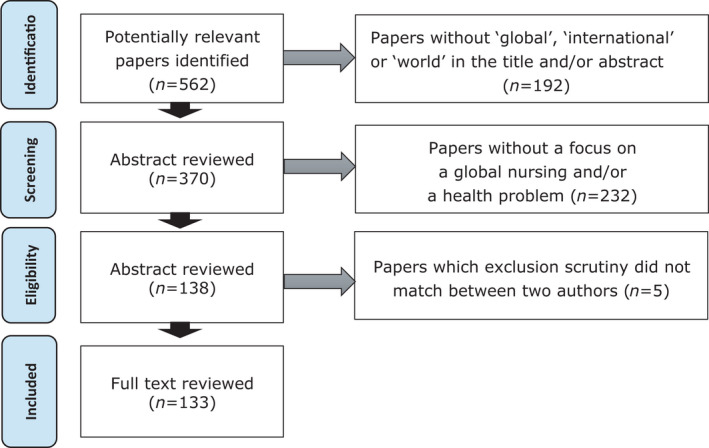
Selection process of papers for review (PRISMA 2009 flow diagram)

There were 562 articles published on 21 June 2019, which were published in international academic journals on nursing or global nursing over the last three years. Among these, 192 articles did not contain “global, international,” or “world” in the title or abstract. We read the abstracts of 370 articles and found that 232 articles did not reflect an awareness of a global problem or research objective in the sampling, data collection or analytical methods. Moreover, five documents did not match the results of the exclusion scrutiny by the two authors. Once these five articles were excluded, 133 articles remained as the final analytical subjects of this study (Figure 1).

### 4.1 Characteristics of the reviewed literature

#### 4.1.1 Countries and regions where authors' affiliated facilities were located

**FIGURE 2:**
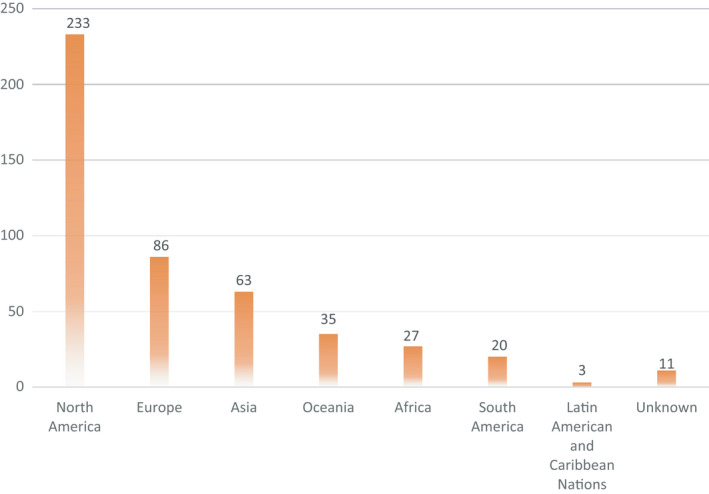
The numbers of authors at each region where their affiliated facilities were located (*N* = 478)

The total number of authors included in this review was 478. For the 11 authors, the countries of their affiliated facilities were not stated. The authors were from 44 countries. The United States (198, 41.4%) was the country with the most authors, followed by the United Kingdom (41, 8.6%), Canada (35, 7.3%) and Australia (26, 5.4%). There were 11 Japanese researchers (2.3%). In terms of regions, North America had the largest number of researchers (233, 48.7%), followed by Europe (86, 18.0%) and Asia (63, 13.2%) (Figure 2).

#### 4.1.2 Types of articles and design

The papers and designs were classified into 20 sub‐categories and then grouped into six categories (essays, practice reports, literature review, quantitative research, qualitative research and mixed method). There were 38 essays (28%), 27 qualitative studies (20%), 25 literature reviews (18%), 24 quantitative studies (18%), 20 practice reports (15%) and one report that used a mixed method (1%). Two out of 133 articles were analytical reports classified under two sub‐categories: a case study and discourse analysis in one case (Evans‐Agnew et al., 2016) and systematic review and meta‐analysis (Tung et al., 2018).

#### 4.1.3 Literature areas

**FIGURE 3:**
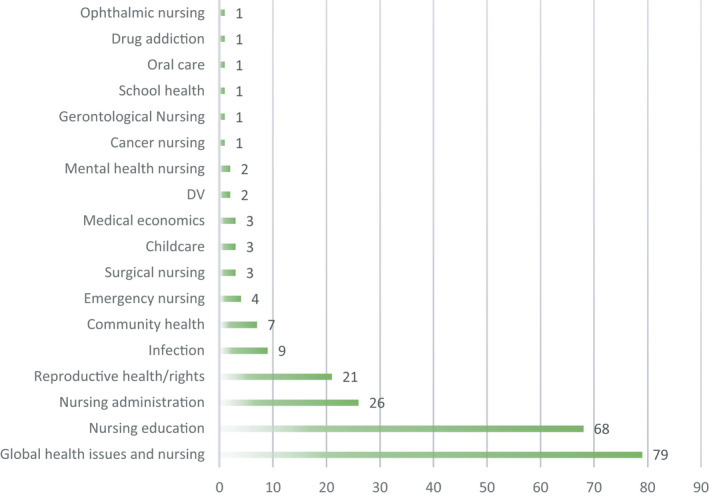
The number of the papers in literature area (*N* = 234*) *This number shows that 45 and 28 papers out of 133 articles have two and three topics, respectively

The reviewed articles were classified into 18 categories. The most commonly mentioned area was “global health issues and nursing” (79 articles, 33.7%), which was followed by “nursing education” (68 articles, 29.1%) and “nursing management” (26 articles, 11.1%) (Figure 3).

### 4.2 Global nursing literature trends

The topics that the literature on global nursing focused on were divided into the following six concepts:

#### 4.2.1 Conceptualization of global nursing

Most articles that attempted to clarify concepts associated with global nursing, such as global health, sustainability and cultural competence (e.g. Cesario, 2017; Goodman, 2016; Milton, 2016; Wilson, Moran, et al., 2016), derived more comprehensive and essential definitions through the comparison of results from previous articles.

For example, in the article by Wilson et al., task force members of the Global Advisory Panel on the Future of Nursing from the Honour Society of Nursing, and Sigma Theta Tau International, qualitatively analysed summaries of 21 articles that sought to establish a definition of global health and six articles on the definition of global nursing. These articles were published in English, Spanish and Portuguese between 2005–2015. In this manner, Wilson et al. derived definitions for global health and nursing. According to their descriptions, global health is related to practices and studies that focus on achieving improvement and equality of health for all people and ensuring the promotion of health and sustainable socio‐cultural, policy and economic systems. The concept of global health denotes global‐scale efforts to improve health that value people's health, animals, the environment and ecosystems (p. 1536). Global nursing was defined as an evidence‐based nursing process that promotes sustainable and global‐scale health and equality. It includes a dedication to ethical practice that requires examining the social determinants of health, such as individual/group care, research, education, leadership, advocacy and policy initiatives, concerning human dignity, human rights and cultural diversity (p. 1537).

#### 4.2.2 Environmental health

Global issues since the 1960s are considered threats to “environmental health.” These issues include climate change, the impacts of extreme weather events, health damage caused by atmospheric pollution, the spread of infectious diseases via water and food, and exposure to ultraviolet rays. The causes of these problems, countermeasures and nurses' roles have been investigated (Divakaran et al., 2016; Goodman, 2016; Griggs et al., 2017; Nicholas & Breakey, 2017; Veenema et al., 2017; Williams & Downes, 2017).

For example, Nicholas and Breakey (2017) performed a literature review of articles, textbooks and environmental health reports. This literature review revealed that nursing organizations had adopted a resolution to counter climate changes on local and global scales and engaged in a leadership role. Nicholas and Breakey pointed out that, for nurses, it is essential to engage in policy discussions about the negative impacts of humans on environmental health. Furthermore, nurses' involvement in countermeasures to combat global warming provides vital opportunities for global leadership. Williams and Downes (2017) described revolutionary courses for nursing students interested in complex humanitarian emergencies (CHEs) and natural disasters. These training pieces include e‐learning courses from the Sphere Project, focusing on nursing accountability and students' quality of humanitarian responses. Further, they provide learning opportunities to obtain the minimum standard that becomes the core of technical support.

#### 4.2.3 Infectious diseases

Many articles have identified global infections and pandemic problems, which have increased in number since the late 1990s. These articles included discussions on nursing practices associated with prevention and response to infections, prevention methods and patient satisfaction. The diseases discussed in these articles included Ebola haemorrhagic fever (Ferranti et al., 2016; Lori et al., 2017; McGillis Hall & Kashin, 2016), Zika fever (Forman et al., 2017; Phillips & Neyland, 2016; Visovsky et al., 2016) and tetanus (Carter & Viveash, 2017).

For example, Lori et al. interviewed 650 women who used birth homes before and after an Ebola haemorrhagic fever outbreak. The results showed that patient satisfaction dropped significantly during the outbreak (pp. 163–171). Forman et al. proposed a useful model that included the prompt practice of prenatal screening at one hospital and a large‐scale and diverse network of community health centres for immigrants. The authors also discussed the importance of localized responses to global outbreaks of Zika fever (pp. 216–224).

#### 4.2.4 Security efforts

Anxiety about international affairs has a significant effect on global nursing practices. There have been several studies and reports on issues such as safety and health issues for immigrants in conflict zones and racism against immigrants (Moyce et al., 2016; Ogunsiji, 2016), human trafficking and organ trade among the poor (Corfee, 2016; Stevens & Berishaj, 2016; Twigg, 2017), and the lack of safety for nurses working in conflict zones (O'Connor, 2017; Taha & Westlake, 2017). These documents focus on the importance of safety and the challenging reality in satisfying the physiological, safety and social needs (affiliation and love) of people, regardless of whether they are patients or nurses. They also emphasized the importance of nurses' roles in such serious situations.

For example, a discussion paper by Corfee (2016) mentions the need to consider the ethical implications for the nursing profession regarding illegal organ trade and organ transplants, known as “transplant tourism.” The article pointed out that the Australian Nursing and Midwifery Federation, the National College of Nursing of Australia and the American Nurses Association have not yet made official statements on transplant tourism positions. The author argues that nursing experts must have a firm stance on health policies and human rights. O'Connor (2017) discussed the importance of warning about the lack of safety for care providers in conflict zones, particularly healthcare specialists' targeting. Healthcare practitioners need to be aware of the risks associated with local armed conflicts. The author also stresses the importance of dealing with the gap in knowledge about the socio‐cultural factors surrounding armed conflicts in the twenty‐first century. Further, the author points out that more focus is needed on the Geneva Convention that stipulates the protection of nursing staff, adherence to international humanitarian laws and global healthcare workers' safety.

#### 4.2.5 Global shortage of nursing personnel

With the global labour shortage and increasing need for health care, many authors have attempted to elucidate measures designed to address problems, such as the shortage of nursing staff and causes for turnover (Kinghorn et al., 2017; Kurth et al., 2016; Leineweber et al., 2016; Liu et al., 2016). This includes socio‐economic and ethical issues associated with transferring nurses to supplement the nursing staff shortage (Moyce et al., 2016; Salami et al., 2016; Shaffer et al., 2018) and deteriorating healthcare situations in one's own country (Efendi et al., 2017).

For example, Salami et al. (2016) described racism against Nigerian nurses who have immigrated to advanced Western nations. They pointed out that countries where nurses migrate to and from must take responsibility for managing their situation as globalization progresses. Efendi et al. (2017) conducted a literature review and showed that Indonesia's healthcare situation was neglected, an exporter of nurses. They also found complex ethical issues associated with Indonesian healthcare workers' migration in the Indonesia–Japan Economic Partnership Agreement and suggested a policy to improve such a situation.

#### 4.2.6 Diversification of study abroad programmes

Many documents included information on modifying study abroad programmes offered as part of classes and exchange programmes in the faculty and graduate school of nursing and assessing their effects. In particular, several articles introduced experiential programmes that allow students to stay in their own country while connecting with people and cultures of other countries in real time via the Internet, without sending students abroad or inviting students from other countries to their own country (Leung et al., 2017; Utley‐Smith, 2017; Wihlborg & Friberg, 2016; Ziemba et al., 2016). Some articles have practised and evaluated an attempt to experience foreign cultures by entering a domestic region with diverse cultural backgrounds for a certain period (Lane et al., 2017; Tanaka et al., 2018). Other programmes have sought to involve students with service learning by engaging them in volunteer work (Brown, 2017; Ryan‐Krause, 2016; Underwood et al., 2016; Wade et al., 2017).

An article entitled “Going Domestic” by Lane et al. (2017) discussed the importance of “studying abroad” in a domestic setting in cities with diverse cultures, based on the active learning strategy pursued by Appalachian State University in New York City. According to Lane et al., because students' cost of studying abroad in developing countries is generally high, only wealthy students may participate. This resulted in a lack of diversity and increased disparity among the students. Because studying abroad takes time, it requires time off, which is difficult for students. It also tends to implant a tourist mindset in students, making it challenging to nurture service‐minded attitudes in the performance of community‐based activities. “Studying abroad” in a domestic setting solves all of these problems and allows students to acquire an open‐minded perspective on culture that transcends their geographic and ethnic experiences (pp. 198–199). Tanaka et al. (2018) also believed that foreign cultures' experiences in their own country enhance cultural understanding among nursing students. The authors surveyed the interests of Japanese nursing students participating in medical health care for foreign residents (MHCFR) in areas with greater ethnic diversity. The results showed that most students thought they would be providing MHCFR in the future.

Nonetheless, they showed little interest in MHCFR and had little knowledge of the accompanying issues. The results also showed that knowledge of MHCFR, recognition of care provisions for foreign residents and contact with foreigners significantly correlated with the level of interest in MHCFR. Tanaka et al. found that it may be adequate to offer experiences to come in contact with foreigners and accept more international students, especially to promote a global health perspective (pp. 41–45).

## 5 DISCUSSION

This review clarified the topics that the paper on global nursing focuses on the conceptualization of global nursing, environmental health, infectious diseases, security efforts, the global shortage of nursing personnel and the diversification of study abroad programmes. Many articles on global nursing dealt with a wide range of serious issues that reflected today's international affairs, labour issues and global environmental problems. Nurses' issues, particularly conflicts and disasters, cannot be adequately addressed by country–country support and cross‐country collaboration.

However, the scope of international nursing research and education in Japan is narrow. Takatsuka and Tanaka (2017) surveyed 24 universities belonging to the Association of Private Nursing Colleges in Japan. They pointed out that the main themes covered in class subjects for international nursing education in Japan were focused on support and care from an international perspective, such as “culture,” “health issues,” “international cooperation,” “developing countries” and “primary care.” In the future, it will be necessary to carry out research and education to tackle healthy environmental problems from a global perspective based on global nursing definitions that address changes over time.

As shown in *global nursing conceptualization*, many articles that tried to elucidate concepts surrounding global nursing practices derived a comprehensive and essential definition based on integrative methods of results from previous studies. This suggests that research in global health and nursing is evolving to a more academically mature stage, from writing researchers' personal views and borrowing definitions from disciplines other than nursing (Meleis, 2018). Wilson, Moran, et al. (2016) defined global nursing as an evidence‐based nursing process that promotes sustainable global‐scale health and equality for all—this is a new definition of nursing in the global era. This suggests that, importantly, nurses provide more practical, research, educational and policy leadership on global health issues and play an influential role in respecting human rights and diversity.

The present study revealed that environmental health was the central theme in practice, education and global nursing research. This demonstrates the seriousness of environmental problems that impact human health and the importance of nursing roles. To achieve a sustainable environment today and in the future, nurses need to warn about how humans live with heavy environmental loads and find improvements. Although global warming is a severe threat to human health, healthcare staff and organizations have been relatively slow to dedicate themselves to sustainable healthcare practices. Griggs et al. (2017) found that “inaction” from “endemic blindness to global issues” and “environmental numbness” is widespread in healthcare organizations. They pointed out that there are many social, cultural and psychological barriers to this issue. It will be necessary to research and clarify measures to overcome these barriers and further educate and raise awareness that creating a sustainable environment is also an essential task of the nursing profession.

Nursing efforts to combat the health hazards of infectious diseases and pandemics are becoming increasingly important in the global community. An example of a worldwide infectious disease was COVID‐19, a known plague from Wuhan, China, in December 2019. Although the virus' significant properties have not yet been elucidated, the effectiveness of public health measures such as social distance, intensive surveillance and tracking of infected individuals has been demonstrated (Mackenzie & Smith, 2020). Nursing professionals need to review many current research findings on prevention and coping with infectious diseases and pandemics in the past and use them for measures against future infections to protect people from the threat of unknown and high‐impact infectious diseases.

Furthermore, this review found that safety assurance problems for both patients and nurses were also significant issues in today's global health. With global situation uncertainty, civilians' safety and health are at risk in conflict zones and in non‐conflict zones due to antisocial activism, such as terrorism. Transplant tourism is also an unacceptable violation of human dignity and rights from nursing and community perspectives. Nurses are expected to enhance their roles in respecting human rights, recovery and promoting health through practice, education and research. O'Connor (2017) showed that health professionals, including nurses, are also close to danger; thus, they will need to share their views on health policy and human rights with the world to ensure their safety and work in a better environment.

Throughout this study, the global debate about the worldwide shortage of nurses has also emerged. Williams and Downes' (2017) efforts, including a course on CHEs and natural disasters, help prepare a nursing workforce that appropriately tackles large‐scale human migrations caused by natural disasters and conflicts. In Japan, education in the national and private graduate school five‐year integrated doctoral programme (Collaborative Disaster Nursing Global Leadership degree programme: DNGL) was initiated in 2014. In 2020, the first global leaders in disaster nursing, with a degree at DNGL, were produced. It is thought that cross‐border international and innovative education will become even more critical in providing a stable supply of the world's nursing workforce in the future.

An essential part of global nursing education is an experiential programme that connects multiple countries and regions online and connects with people of different cultures in real time. We discovered that many universities and organizations are implementing such programmes and achieving results in the present study. These flexible programmes that are being developed and implemented allow students to experience foreign cultures while living in their own countries. This experience is integrated with service learning for practical skills. As Lane et al. (2017) discussed, studying abroad usually incurs a high cost, limiting the participating students to affluent backgrounds and creating a disparity between students. Therefore, the Internet's use is necessary to “study abroad” in a domestic setting and facilitate educational efforts that integrate service learning.

Today, significant changes in the environment change ecosystem balance and affect people's lifestyles and working styles. Nursing professionals in the 21st century will need new strategies and models to create and maintain a healthy environment (Meleis, 2018) and health care for individuals and groups. The development of some types of environment‐related theories that guide the development of a healthy environment and behaviour for environmental changes and healthcare policies (Salazar & Primomo, 1994) through many existing research findings will be required in the future.

### 5.1 Limitations and future directions

The results of this review should be considered with some limitations. The most significant limitation was that the search databases used in this review were limited to PubMed. Furthermore, the articles reviewed in this study were limited to those published between 2016–2018. Therefore, it was impossible to show the research results, including trends in research on COVID‐19. Since COVID‐19 is considered to significantly impact nursing practice, research and education, examining research trends after 2020 is a fundamental challenge for the future.

### 5.2 Conclusion

Global nursing's latest trends reflect today's severe international health problems, labour issues and global environmental affairs. Based on these global nursing topics, it is necessary to develop new strategies, nursing models and environment‐related theories to create and maintain a healthy environment.

## CONFLICT OF INTEREST

There are no conflicts of interest relating to this article.

## AUTHOR CONTRIBUTION

Each author of this study has substantially contributed to conducting the underlying research and drafting the manuscript. The first author, Yatsu, contributed to the conception and design of this study, data collection, data analysis and interpretation, manuscript writing and final approval of the version to be submitted. The second author, Saeki, performed the data collection, data analysis and interpretation, revising the manuscript critically and approval of the final version.

## Data Availability

The data that support the findings of this study are openly available at https://docs.google.com/spreadsheets/d/1fyXluSaoKv5bE3412nn4tBxQ2CeZ0hhzWOxnL4hc8M8/edit#gid=1890610693.

## APPENDIX 1

### Category list

**Table:**

Concepts (topics)	Categories (themes)	Codes	Literature
Conceptualization of global nursing	The concepts of global nursing are clarified by integrative methods of previous research on global nursing practices	Clarification of global health	Clark et al. (2016), Cesario (2017), Edmonson et al. (2017), Evans‐Agnew et al. (2016), Gennaro et al. (2016), Gimbel et al. (2017), Wilson, Mendes, et al. (2016), Wilson, Moran, et al. (2016)
		Clarification of global nursing	Gimbel et al. (2017), Luyben et al. (2017), Wilson, Moran, et al. (2016)
		Clarification of sustainability	Benton & Shaffer (2016), Collins et al. (2018), Goodman (2016), Griggs et al. (2017)
		Clarification of global alliance	Gresh et al. (2017)
		Cultural competencies of global health nursing	Cantey et al. (2017), Cruz et al. (2017), Milton (2016), Warren et al. (2016)
Environmental health	Environmental health is the central theme of literatures on global nursing.	Impacts of climate change and extreme weather events	Divakaran et al. (2016), Nicholas and Breakey (2017), Goodman (2016), Griggs et al. (2017), Veenema et al. (2017), Williams and Downes (2017)
		Health damage caused by atmospheric pollution	Nicholas and Breakey (2017)
		Spread of infectious diseases via water and food	Nicholas and Breakey (2017), Veenema et al. (2017)
		Exposure to ultraviolet rays	Nicholas and Breakey (2017)
		Complex humanitarian emergencies (CHEs) and natural disasters	Williams and Downes (2017)
Infection diseases	There are many articles on nursing interventions associated with the prevention, treatment and spread of infectious diseases	Ebola haemorrhagic fever	Edmonson et al. (2017), Ferranti et al. (2016), Lori et al. (2017), McGillis Hall and Kashin (2016)
		Zika fever	Forman et al. (2017), Phillips and Neyland (2016), Visovsky et al. (2016)
		Tetanus	Carter and Viveash (2017)
		Global infectious diseases and vaccinations	Edmonson et al. (2017), Macintosh et al. (2017)
Security efforts	Efforts for safety of refugees and healthcare workers are considered important in nursing	Safety and health issues for immigrants in conflict zones and racism against immigrants	Al‐Natour et al. (2016), Forman et al. (2017), Moyce et al. (2016), Ogunsiji (2016), Sevinç et al. (2016)
		Human trafficking and organ trade among the poor	Corfee (2016), Edmonson et al. (2017), Fraley & Aronowitz (2017), Scannell et al. (2018), Stevens and Berishaj (2016), Twigg (2017)
		Lack of safety for nurses working in conflict zones	O'Connor (2017), Taha and Westlake (2017)
Global shortage of nursing personnel	The focus is on the lack of a global nursing staff	Shortage of nursing staff and causes for turnover	Kinghorn et al. (2017), Kurth et al. (2016), Leineweber et al. (2016), Liu et al. (2016)
		Socio‐economic and ethical issues associated with transferring nurses to supplement the nursing staff shortage	Lum et al. (2016), Mikkonen et al. (2016), Moyce et al. (2016), Newton et al. (2016), Salami et al. (2016), Shaffer et al. (2018)
		Deterioration of healthcare situation in home country	Efendi et al. (2017)
		Investing in global labour development	Downing et al. (2016), Floyd & Brunk (2016), Krubiner et al. (2016), Kurth et al. (2016), Salmon & Maeda (2016), Spies et al. (2017)
		Global leader placement and partnership	Bryant‐Lukosius et al. (2017), Ferguson et al. (2016), Gower et al. (2017), Holland & Magama (2017), Rosser et al. (2017), Rumsey et al. (2017), Shin et al. (2016), Stone et al. (2016)
Diversification of study abroad programmes	International study programmes incorporate flexible methods, such as foreign culture experience within one's own country, integration with service learning and joint online programmes with other countries.	Experiential programmes that allow students to stay in their own country while connecting with people and cultures of other countries in real time via the Internet	Falleiros de Mello et al. (2018), Leung et al. (2017), Utley‐Smith (2017), Wihlborg and Friberg (2016), Ziemba et al. (2016)
		Attempt to enter a region with diverse cultural backgrounds in the country for a certain period of time to promote cross‐cultural experiences	Lane et al. (2017), Tanaka et al. (2018)
		Efforts to have students engage in volunteer work with service‐learning functions	Brown (2017), Ryan‐Krause (2016), Underwood et al. (2016), Wade et al. (2017)
		Strategic global partnerships and global health programmes	Chan et al. (2018), Currie et al. (2018), Garrido et al. (2016), Lake et al. (2017), Napolitano & Duhamel (2017), Parfitt & Nahar (2016), Stringer et al. (2016)

## APPENDIX 2

### The number and ratio of authors per country in each region (*N* = 478)

**Table:**

Region	Number and % of authors		Country	Number of authors
North America	233	48.70%	United States	198
			Canada	35
Europe	86	18%	United Kingdom	41
			Sweden	13
			Ireland	9
			Finland	7
			Italy	4
			Turkey	4
			Denmark	3
			Switzerland	2
			Belgium	2
			Germany	1
Asia	63	13.20%	China	12
			Japan	11
			Korea	7
			India	6
			Kuwait	6
			Saudi Arabia	5
			Singapore	3
			Cambodia	2
			Laos	2
			Bangladesh	2
			United Arab Emirates	2
			Jordan	2
			Thailand	1
			Vietnam	1
			Indonesia	1
Oceania	35	7.40%	Australia	26
			New Zealand	9
Africa	27	5.60%	Botswana	6
			Malawi	5
			Rwanda	4
			Ethiopia	4
			South Africa	3
			Uganda	2
			Kenya	1
			Tanzania	1
			Nigeria	1
South America	20	4.2%	Brazil	12
			Nicaragua	5
			Mexico	3
Latin American and Caribbean Nations	3	0.6%	Haiti	1
			Dominica	1
			The Bahamas	1
Unknown	11	2.3%		11

## APPENDIX 3

### The number and ratio of papers by design (*N *= 135^a^)

**Table:**

Types of articles	Number and % of papers		Design of articles	Numbers of papers
Essay	38	28.2%	Discussion paper	31
			Theoretical paper	4
			Position paper	2
			Philosophical paper	1
Qualitative research	27	20%	Descriptive	13
			Phenomenological/hermeneutic	4
			Grounded theory approach	2
			Concept analysis	2
			Discourse analysis	2
			Case study	2
			Ethnography	1
			Feminist critique	1
Literature study	25	18.5%	Literature review	15
			Systematic review	10
Quantitative research	24	17.8%	Descriptive	17
			Quasi‐experiment	3
			Before‐after analysis	3
			Meta‐analysis	1
Practical report	20	14.8%		20
Mixed method	1	0.7%		1

^a^Two papers have duplicative research design.
